# Association of retinal vessel pathology and brain atrophy in relapsing-remitting multiple sclerosis

**DOI:** 10.3389/fimmu.2023.1284986

**Published:** 2023-11-28

**Authors:** Eva Feodora Romahn, Tun Wiltgen, Matthias Bussas, Lilian Aly, Rebecca Wicklein, Christina Noll, Achim Berthele, Vera Dehmelt, Christian Mardin, Claus Zimmer, Thomas Korn, Bernhard Hemmer, Jan S. Kirschke, Mark Mühlau, Benjamin Knier

**Affiliations:** ^1^ Department of Neurology, Klinikum rechts der Isar, TUM School of Medicine and Health, Technical University of Munich, Munich, Germany; ^2^ Department of Ophthalmology, University Hospital of Erlangen-Nuremberg, Erlangen, Germany; ^3^ Department of Neuroradiology, Klinikum rechts der Isar, TUM School of Medicine and Health, Technical University of Munich, Munich, Germany; ^4^ Institute for Experimental Neuroimmunology, Klinikum rechts der Isar, TUM School of Medicine and Health, Technical University of Munich, Munich, Germany; ^5^ Munich Cluster for Systems Neurology (SyNergy), Munich, Germany

**Keywords:** optical coherence tomography, optical coherence tomography angiography, multiple sclerosis, magnetic resonance imaging, brain atrophy, disability

## Abstract

**Background:**

Optical coherence tomography angiography (OCTA) allows non-invasive assessment of retinal vessel structures. Thinning and loss of retinal vessels is evident in eyes of patients with multiple sclerosis (MS) and might be associated with a proinflammatory disease phenotype and worse prognosis. We investigated whether changes of the retinal vasculature are linked to brain atrophy and disability in MS.

**Material and methods:**

This study includes one longitudinal observational cohort (n=79) of patients with relapsing-remitting MS. Patients underwent annual assessment of the expanded disability status scale (EDSS), timed 25-foot walk, symbol digit modalities test (SDMT), retinal optical coherence tomography (OCT), OCTA, and brain MRI during a follow-up duration of at least 20 months. We investigated intra-individual associations between changes in the retinal architecture, vasculature, brain atrophy and disability. Eyes with a history of optic neuritis (ON) were excluded.

**Results:**

We included 79 patients with a median disease duration of 12 (interquartile range 2 - 49) months and a median EDSS of 1.0 (0 - 2.0). Longitudinal retinal axonal and ganglion cell loss were linked to grey matter atrophy, cortical atrophy, and volume loss of the putamen. We observed an association between vessel loss of the superficial vascular complex (SVC) and both grey and white matter atrophy. Both observations were independent of retinal ganglion cell loss. Moreover, patients with worsening of the EDSS and SDMT revealed a pronounced longitudinal rarefication of the SVC and the deep vascular complex.

**Discussion:**

ON-independent narrowing of the retinal vasculature might be linked to brain atrophy and disability in MS. Our findings suggest that retinal OCTA might be a new tool for monitoring neurodegeneration during MS.

## 1 Introduction

Though the retina reflects an unmyelinated part of the central nervous system (CNS), it is frequently affected by different autoimmune diseases. Atrophy of the retinal nerve fiber layer (RNFL) and ganglion cells is frequently found in most patients with multiple sclerosis (MS) and has been linked to a worse disease prognosis (1–4). Moreover, ganglion loss over time might reflect CNS atrophy and is discussed as a new biomarker for neurodegenerative processes in the course of MS (5, 6).

Optical coherence tomography (OCT) angiography (OCTA) allows for non-invasive, and high-resolution imaging of retinal blood vessels. Originally developed as new imaging modality in ophthalmology, OCTA has increasingly been applied in patients with autoimmune diseases of the CNS during the last years. Recent research suggests that patients suffering from CNS autoimmune diseases also display alterations of the retinal vasculature. Though different OCTA devices have been applied to study retinal vessel pathology in MS, a rarefication of superficial retinal vessel structures has been reported in most studies (7). Superficial retinal vessel loss is evident during the first 90 days after acute optic neuritis (ON) and linked to a poor visual acuity (8–10). We could recently show that narrowing of superficial retinal vessels also occurs in eyes without a history of ON, might be associated with a pro-inflammatory intrathecal immunity, and might predict a worse disease course in patients with early relapsing-remitting MS (RRMS) (11). These data suggest that the retinal vasculature might be involved in CNS autoimmunity. The underlying pathophysiology is not yet understood. It is unclear whether rarefication of the retinal vasculature occurs secondary to metabolic effects of retinal neurodegeneration or whether retinal vessels or the blood-retinal-barrier are primarily affected during MS (10).

In the current study, we evaluated whether alterations of retinal vessel structures are linked to brain atrophy and qualify as a biomarker for CNS neurodegeneration in patients with RRMS.

## 2 Materials and methods

### 2.1 Study design

The current study includes a longitudinal observational cohort of patients with clinically isolated syndrome (CIS) and RRMS according to the McDonald criteria 2017 between 18 and 75 years of age (12). We retrospectively selected patients from a running prospective observational cohort study of patients with early MS who have been enrolled between 2018 and 2022 at the Department of Neurology, Klinikum rechts der Isar of the Technical University of Munich. We selected patients with CIS and early RRMS who received at least two OCT, OCTA, and brain MRI examinations during a follow-up duration of at least 18 months. At baseline, patients underwent a thorough clinical assessment with evaluation of the expanded disability status scale (EDSS), the symbol digit modalities test (SDMT), the timed 25-foot walk test (T25W) and the 9-hole peg test (9-HPT). OCT, OCTA, and brain MRI were performed. We took a detailed medical history in all patients. Baseline visits were scheduled at least 30 days after the last clinical relapse. All patients received an assessment of the EDSS, SDMT, T25W, 9-HPT, a retinal OCT, an OCTA scan and a cerebral MRI scan once per year at pre-defined timepoints (all 12 months). Additional clinical visits with assessment of the EDSS occurred depending on clinical conditions of the respective patients. All of these examinations and procedures were performed within a maximum period of 14 days. Immunotherapies were categorized as disease-modifying therapy (DMT) class 1 (glatiramer acetate, interferon beta-1a/b, dimethyl fumarate, teriflunomide), class 2 (fingolimod, ozanimod, cladribine) and class 3 (natalizumab, rituximab, ocrelizumab, Ofatumumab, alemtuzumab), and “no DMT” if patients were not set on any immunotherapy. Disability worsening as measured by the EDSS was defined if an increase of the EDSS confirmed at least 3 months after the first documentation occurred during the study follow-up (EDSS increase of ≥ 1.5 if the baseline EDSS was 0, increase of ≥ 1.0 if the baseline EDSS was between 1.0 and 5.0). We defined worsening of the SDMT if a reduction of at least 4 points occurred (confirmed in a second follow-up visit) (13) and worsening of the T25W and 9HPT if an ≥ 20% increase in test times were recognized (confirmed in a second follow-up visit) (14). We excluded patients with any relevant eye disease possibly affecting the integrity of the retina and a refractory errors > 6 dioptres. Eyes with a history of ON, suspected subclinical ON (as delineated below), or poor OCTA quality were removed from the analysis as well. We furthermore removed patients from the analysis if exclusion of single eyes resulted in missing OCTA data on both eyes at any timepoint.

In the first step, we aimed to reproduce already published associations of longitudinal retinal neurodegeneration and brain atrophy (5). In the second step, we searched for a possible relationship of longitudinal changes of the retinal vasculature and brain volume loss. In the third step, we analyzed whether changes of retinal vessel structures are linked to clinical measures of disability.

### 2.2 Standard protocol approvals, registrations, and patient consents

STROBE guidelines were followed for reporting of observational studies. The ethics committee of the Technical University of Munich, School of Medicine evaluated and approved the current study (116/16 S, 9/15 S). We adhered to the Declaration of Helsinki and all participants gave written informed consent.

### 2.3 OCT and OCTA analysis

We accomplished conventional retinal OCT images as described previously (15) and included examination of the peripapillary RNFL (pRNFL) and the macula (30° x 25° macular scan). All scans were thoroughly checked for sufficient image quality according to the OSCAR-IB criteria (16). Segmentation of retinal layers was done automatically by a manufacturer provided software (Eye Explorer, v2.5.4.) and was manually corrected if necessary.

We acquired OCTA examinations on both eyes of each patient under low-lighting conditions (Heidelberg Spectralis OCT 2 with angiography module, Heidelberg Engineering) by experienced operators as described elsewhere (10, 11). In brief, en face images were recorded (2.9 × 2.9 mm scanning area) manually centred on the fovea centralis and with active eye tracking. We performed segmentation of the macular area into the superficial (SVC) and the deep vascular complex (DVC) using a manufacturer derived algorithm (v2.5.4). We used the Erlangen Angio Tool (17) for quantification of retinal vessel densities as described elsewhere (10, 11). Vessel structures were assessed within a centred perifoveal circle between a radius of 0.8 mm and 2.9 mm (area 6.1 mm²). The size of the foveal avascular zone (FAZ) was calculated using a MatLab-based software tool (MathWorks, vR2019b). To ensure sufficient OCTA image quality, we checked all OCTA scans according to the OSCAR-MP criteria (18).

As an established standard procedure, eyes with a history of clinical and/or unilateral subclinical ON were excluded from the analyses. We assumed a history of subclinical ON if a difference of both the pRNFL and the GCIP of more than 5 and 4 µm, respectively, was present between both eyes (19). We followed the APOSTEL 2.0 recommendations for reporting quantitative OCT studies (20).

### 2.4 Cerebral MRI acquisition and processing

We recorded all cerebral MRI scans by using the same 3 Tesla scanner (Achieva, Philips Healthcare) with the identical protocol. All MRI examinations were performed during daytime (7 AM to 6 PM). We acquired three-dimensional T1- and T2‐weighted fluid‐attenuated inversion recovery (FLAIR) images as described elsewhere (2). All MRI examinations were further processed with the software packages SPM12, CAT12 (https://www.neuro.uni-jena.de/cat12/) and LST (https://www.applied-statistics.de/). Lesions of the white matter were segmented from FLAIR and T1-weighted images by the “lesion growth algorithm” (21). Brain volumes were calculated by the sum of the brain white and grey matter. Cortical thickness was taken from CAT’s surface-based maps (22). We applied a reverse brain mask approach to assess the total intracranial volume (23). We extracted regional brain volumes from grey matter images with the LONI Probabilistic Brain (LPBA40) atlas (24) for putamen and neuromorphometrics atlas (25) for thalamus. All images were visually inspected for MRI artifacts by experienced operators.

### 2.5 Statistical analysis

Statistical analyses was done using GraphPad Prism (v9.3.1) software. To account for inter-eye correlations of OCT and OCTA measures, we used mean values of both eyes as one single data point when both eyes were available for calculation. If one eye was excluded due to our exclusion criteria (see above), we used the values of the remaining eye as described elsewhere (2). For evaluation of longitudinal alterations of OCT and OCTA measures, eyes with a history of clinical ON, suspected subclinical ON, or poor image quality at baseline or during the study follow-up were removed from this analysis for every timepoint completely. To quantify longitudinal annual changes of OCT, OCTA, and MRI measures, we performed least square linear regression analyses. Here, the best-fit value of the slope between OCT/OCTA/MRI time points (referenced to baseline, x-axis) and the respective measurements (y-axis) was calculated. This value reflects the linear change of the respective measure per time interval (year). We applied both univariate simple and multiple linear regression models to search for associations between longitudinal OCT or OCTA measures, changes of different brain volumes and disability as already described by others (5). We corrected all multiple linear regression models for the covariates age, sex, total intracranial volume, and disease duration, if not otherwise stated (11). We furthermore performed a Bonferroni correction for multiple comparison. To evaluate whether relevant longitudinal changes of OCT, OCTA, and MRI variables occurred, we applied a paired t-test (if normally distributed) or the Wilcoxon matched-pairs signed rank test (if not normally distributed) of the baseline and the respective last follow-up measure. To test for differences of longitudinal OCT and OCTA measures between patients with EDSS, SDMT, T25W, 9HPT worsening and those without, we used an unpaired t test or a non-parametric Mann–Whitney U test depending on normal distribution. Values are described as mean ± standard deviation (SD) (if normally distributed) or otherwise as median (25%-75% interquartile range). The statistical significance threshold was p<0.05.

## 3 Results

### 3.1 Study cohort and baseline characteristics

A total of 136 patients were identified and included into the current study. Fifty-seven patients were excluded due to the exclusion criteria whereas 42 patients revealed a history of ON in one eye and poor OCTA quality on the remaining eye at any study visit and 15 patients had poor OCTA quality on both eyes. Thus, we included 79 patients with a median age of 38 (32 - 47) years and a disease duration of 12 (2 - 49) months into the final analysis. Patients were only mildly clinical affected (median EDSS 1.0 [0 - 2.0]) (**Table 1**). We excluded 26 eyes due to a history of clinical ON, 2 eyes due to subclinical ON and another 9 eyes due to poor OCTA quality. In total, 121 eyes of 79 patients included in the study. Twelve patients from this cohort have already been reported in other studies (10, 11).

**Table 1:** Clinical characteristics, OCT, OCTA and brain MRI measures at baseline.

	Patient cohort (n=79)
Clinical parameters	
Age, years	38 (32 -47)
Female sex, No. (%)	53 (67)
Disease duration, months	12 (2 – 49)
EDSS	1.0 (0 – 2.0)
SDMT, score	63 (56 – 69)
T25W, seconds	4.4 (3.9 – 4.7)
9-HPT dominant hand, seconds	17.8 (16.5 – 19.8)
9-HPT non-dominant hand, seconds	18.9 (17.4 – 21.3)
Diagnosis, No. (%) - CIS - RRMS	4 (5) 75 (95)
Disease modifying therapy, No. (%) - no therapy - category 1 - category 2 - category 3	10 (13) 40 (51) 16 (20) 13 (16)
Retinal OCT	
pRNFL, µm	98 (89 – 106)
GCIP, µm	69 (66 – 73)
INL, µm	34 (33 – 36)
Retinal OCTA	
SVC, % vessel density	26 (24 – 28)
DVC, % vessel density	26 (25 – 28)
FAZ, mm²	0.22 (0.16 – 0.28)
Cerebral MRI	
Total intracranial volume, ml	1133 (1068 – 1221)
White matter, ml	490 (456 – 548)
Grey matter, ml	633 (599 – 664)
Grey matter thalamus, ml	10.2 (9.4 – 10.9)
Grey matter putamen, ml	9.0 (8.3 – 9.8)
Cortical thickness, mm	2.6 (2.5 – 2.6)
Lesion volume, ml	1.4 (0.6 – 6.1)
Number of lesions	14 (7 – 22)

Values are provided as median (25% - 75% interquartile range); expanded disability status scale (EDSS), symbol digit modalities test (SDMT), timed 25-foot walk (T25W), 9-hole peg test (9-HPT), peripapillary retinal nerve fiber layer (pRNFL), combined ganglion cell and inner plexiform layer (GCIP), inner nuclear layer (INL), superficial (SVC) and deep vascular complex (DVC), foveal avascular zone (FAZ).

### 3.2 Longitudinal associations of the retinal architecture, vasculature, and brain volumes

Patients from our longitudinal observational cohort underwent a median of 3 (2-3) study visits over a median follow-up duration of 728 (714-742) days. Patients showed thinning of the pRNFL and the GCIP as well as rarefication of vessel structures within the SVC (**Table 2**). Moreover, we saw a reduction of the total intracranial volume and the cortical thickness accompanied by white and grey matter loss, in particular of the thalamus and putamen (**Table 2**). We observed a trend toward an increase in T2 lesion volume while the number of lesions remained unchanged (**Table 2**). DMT exposure during the study follow-up did not significantly affect the rates of OCT, OCTA and brain MRI measures (data not shown). A total of 9 patients (12% of patients) suffered from confirmed EDSS worsening, 9 patients (12%) from SDMT worsening, 5 patients (6%) from T25W worsening, two patients (3%) from 9-HPT worsening of the dominant hand one patient (1%) from worsening of the non-dominant hand during the follow-up.

**Table 2:** Longitudinal alterations of the retinal architecture, vasculature and brain volumes.

	Annualized change	p-value
Retinal OCT changes		
pRNFL, µm per year	-0.48 (-0.97 – 0.30)	**0.004**
GCIP, µm per year	-0.18 (-0.53 – 0.00)	**0.0003**
INL, µm per year	0.00 (-0.01 – 0.01)	0.17
Retinal OCTA changes		
SVC, % vessel density per year	-0.18 (-0.68 – 0.08)	**0.03**
DVC, % vessel density per year	-0.08 (-0.56 – 0.51)	0.48
FAZ, mm² per year	0.001 (-0.006 – 0.009)	0.31
Brain MRI changes		
Total intracranial volume, ml per year	-4.2 (-6.2 – -2.1)	**<0.0001**
White matter, ml per year	-1.9 (-3.5 – -0.4)	**<0.0001**
Grey matter, ml per year	-3.3 (-5.8 – 0.2)	**<0.0001**
Grey matter thalamus, ml per year	-0.04 (-0.12 – 0.05)	**0.02**
Grey matter putamen, ml per year	-0.06 (-0.10 – 0.00)	**0.0001**
Cortical thickness, mm per year	-0.004 (-0.013 – 0.004)	**0.005**
Lesion volume, ml per year	0.03 (-0.09 – 0.33)	0.054
Number of lesions, change per year	0.0 (-0.5 – 1.0)	0.65

Values are provided as median (25% - 75% interquartile range); clinically isolated syndrome (CIS), relapsing remitting multiple sclerosis (RRMS), peripapillary retinal nerve fiber layer (pRNFL), combined ganglion cell and inner plexiform layer (GCIP), inner nuclear layer (INL), superficial (SVC) and deep vascular complex (DVC), foveal avascular zone (FAZ); p-value indicates the result of a paired t-test or Wilcoxon matched-pairs signed rank test of baseline and last follow-up measures; statistically significant p-values are marked in bold.

Longitudinal changes of the GCIP thickness were by trend linked to alterations of the SVC (ΔGCIP ~ ΔSVC: β = 0.10 [95% CI -0.03 to 0.23], p=0.13). No linkage was seen between the remaining OCT and OCTA measures (data not shown).

**Figure 1:**
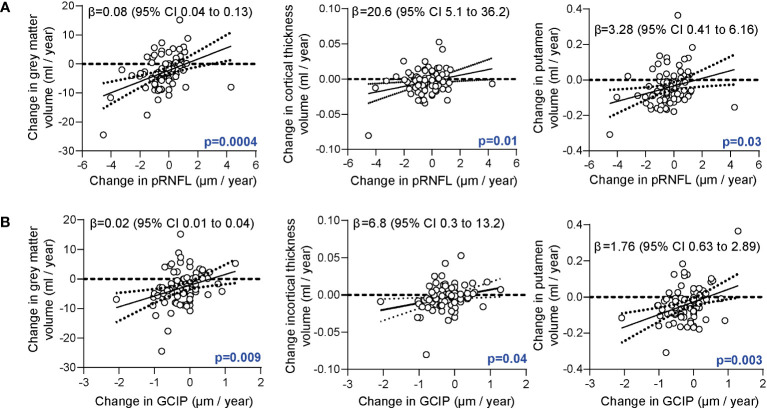
Association of the retinal architecture and brain atrophy. **(A)** Correlation of annualized changes of the peripapillary retinal nerve fiber layer (pRNFL) thickness and annualized changes of total grey matter volume, cortical thickness, and grey matter volume of the putamen. **(B)** Correlation of annualized changes of the combined ganglion cell and inner plexiform layer (GCIP) thickness and changes of total grey matter volume, cortical thickness and volume of the putamen. **(A, B)** Multiple linear regression models corrected for age, sex, disease duration, total intracranial volume; β estimates (line) and 95% confidence interval (CI; dotted lines).

In the first step we searched for relationships between retinal neurodegeneration and brain atrophy. As expected (5), retinal axonal and ganglion cell loss were linked to grey matter atrophy, cortical atrophy, and volume loss of the putamen (**Figures 1A, B**).

**Figure 2:**
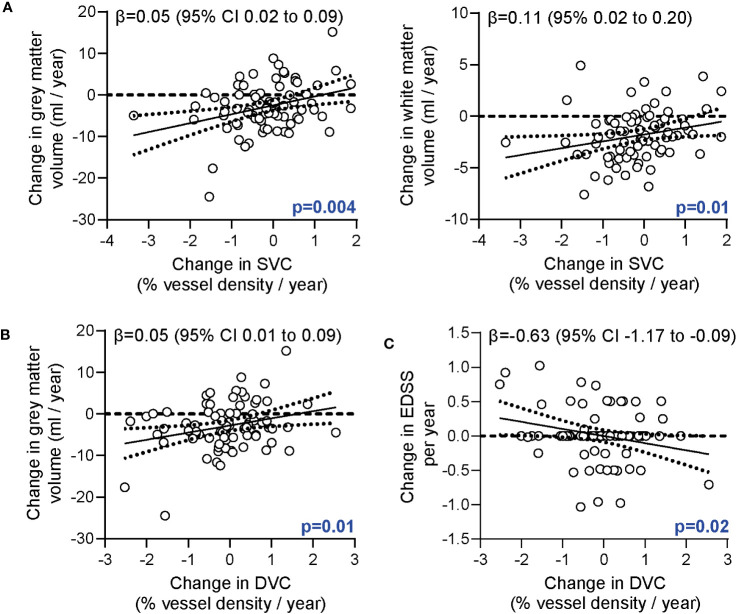
Association of the retinal vasculature, brain atrophy and disability. **(A)** Association of annualized changes of vessel densities of the superficial vascular complex (SVC) and annualized changes of the grey and white matter volumes. **(B)** Association of annualized changes of vessel densities of the deep vascular complex (DVC) and annualized changes of the grey matter volume. **(C)** Correlation of annualized changes of vessel densities of the DVC and annualized changes of the EDSS**. (A, B)** Multiple linear regression models corrected for age, sex, disease duration; **(C)** Multiple linear regression models corrected for age, sex, disease duration, total intracranial volume; β estimates (line) and 95% confidence interval (CI; dotted lines).

In the second step, we analyzed associations of longitudinal alterations of the retinal vasculature and brain volumes. Here, retinal vessel rarefication of the SVC was associated with both grey and white matter atrophy (**Figure 2A**). This association remained robust when additionally correcting for longitudinal changes of GCIP (ΔSVC ~ Δ grey matter + ΔGCIP: β = 0.04 [95% CI 0.01 to 0.08], p=0.02; ΔSVC ~ Δ white matter + ΔGCIP: β = 0.10 [95% CI 0.01 to 0.19], p=0.04). Moreover, retinal vessel loss of the DVC was associated with grey matter atrophy (**Figure 2B**). This linkage remained robust when correcting for GCIP loss (ΔDVC ~ Δ grey matter + ΔGCIP: β = 0.05 [95% CI 0.01 to 0.09], p=0.01). No linkage between changes in the FAZ and brain volumes or white matter lesion estimates was recognized (data not shown).

In the third step, we searched for possible relations of retinal vessel changes and disability. Here, intra-individual rarefication of deep vessel structures during the study follow-up was associated with an increase in EDSS values (**Figure 2C**). This finding remained robust when additionally correcting for longitudinal changes of the GCIP (ΔDVC ~ Δ EDSS + ΔGCIP β = -0.63 [95% CI -1.17 to -0.08], p=0.03). We did not recognize any association of longitudinal retinal vessel loss and annualized alterations of the SDMT, T25W or 9-HPT.

**Figure 3:**
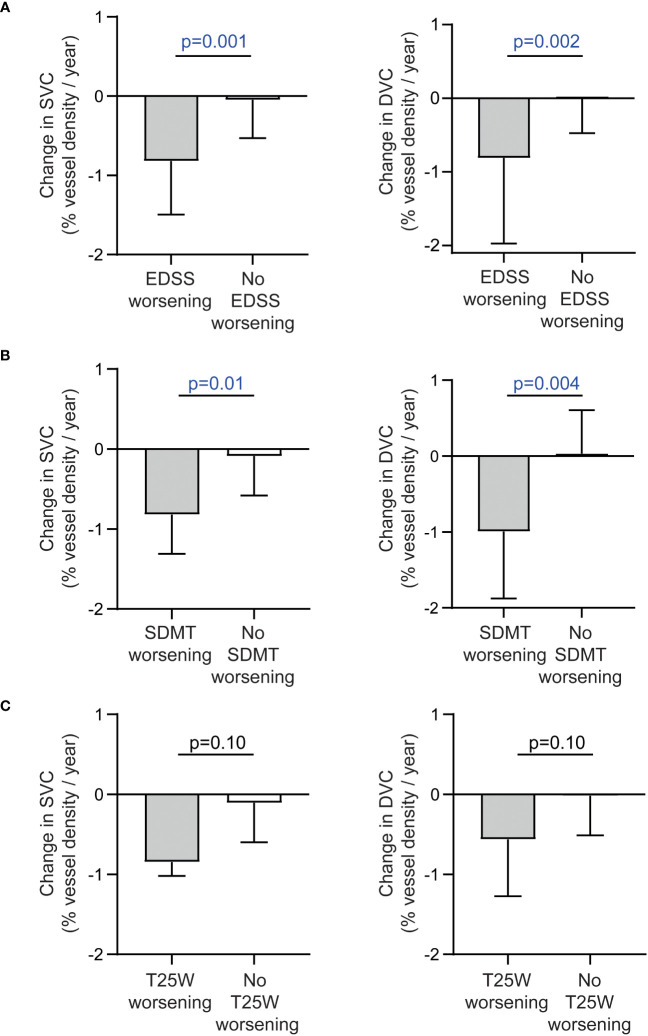
Alteration of the retinal vasculature in patients with and without disability worsening. **(A)** Annualized changes of vessel densities of the superficial (SVC) and deep vascular complex (DVC) in patients with and without confirmed worsening of the expanded disability status scale (EDSS). **(B)** Annualized changes of vessel densities of the SVC and DVC in patients with and without confirmed worsening of the symbol digit modalities test (SDMT). **(C)** Annualized changes of vessel densities of the SVC and DVC in patients with and without confirmed worsening of the timed 25-foot walk test (T25W)**. (A-C)** Unpaired t test or non-parametric Mann–Whitney U test (depending on normal distribution); bars reveal median values and the 75% measure of the 25%-75% interquartile range.

Patients with confirmed EDSS worsening revealed a pronounced thinning of retinal vessel structures of the SVC and DVC as compared to those with stable EDSS values (**Figure 3A**). Moreover, patients with SDMT and by trend T25W worsening showed higher rates of SVC and DVC vessel loss as compared to those with stable measures (**Figures 3B, C**). No significant differences were recognized in longitudinal alterations of the FAZ, pRNFL and GCIP in patients with or without worsening of the EDSS, SDMT and T25W. Due to the low sample sizes of patients with 9-HPT worsening, we did not perform additional statistical tests here.

Taken together, our data suggest that individual changes in the retinal vasculature might reflect central nervous system neurodegeneration during the disease course of MS.

## 4 Discussion

In the current study, we aimed to integrate changes of retinal vessels into the pathophysiological concept of MS. Our findings indicate that the rarefication of the retinal vasculature might be linked to CNS neurodegeneration and disability. Therefore, OCTA based analysis of retinal vessels may add novel aspects to the clinical and therapeutical approach of patients with early MS.

Retinal vessel loss as measured by OCTA is frequently found in the eyes of individuals with MS and past ON. Here, rarefication of superficial vessel structures after ON is a consistent finding across different OCTA devices (7–11, 26). We could recently show that superficial vessel thinning evolves simultaneously to GCIP atrophy after acute ON (10). Some studies, and the majority by using the Optovue OCTA device, also detected decreased vessel densities of the (peri-)foveal DVC in eyes with an ON history (7, 9, 27) whereas other groups using the Heidelberg Engineering Spectralis did not find any alterations of the DVC during MS (8, 10, 11, 28). However, there is growing literature suggesting that retinal vessel pathology also occurs in eyes without past ON during MS (8, 11, 28–30).

In our study, alterations of the retinal vasculature were linked to clinical measures of disability. Different cross-sectional studies also described an association between decreased retinal vessel densities and increased measures of disability like the EDSS or Multiple Sclerosis Functional Composite (MSFC) (8, 30). In our study, decreasing DVC vessel densities were associated with increasing EDSS values in the longitudinal cohort. In line with this, Lanzillo et al. found an association of decreasing parafoveal vessel densities (without differentiating into SVC or DVC) and increasing EDSS values after a 1-year prospective follow-up in patients with MS (31). These data suggest that (deep) retinal vessel loss might reflect disability progression during MS.

In the present study, retinal axonal and ganglion cell atrophy were associated with grey matter loss and cortical atrophy. There is substantial evidence in the literature that both retinal ganglion cell and axonal loss reflect CNS neurodegeneration during MS. By applying 7T MRI imaging, Sinnecker et al. could show that a focal optic radiation damage results in consecutive atrophy of corresponding RNFL thinning of the retina suggesting that retrograde trans-synaptic neurodegeneration occurs during MS (32). Other studies suggested that retrograde trans-synaptic neurodegeneration even applies for focal lesions outside the visual pathway. Longitudinal intra-individual ganglion cell loss has been linked to whole-brain and especially grey matter atrophy in patients with RRMS by both longitudinal retrospective and prospective studies (5, 33). Moreover, different cross-sectional studies provided evidence that retinal ganglion cell loss might also be associated with spinal cord atrophy in individuals with MS (34, 35). These data suggest that retinal ganglion cell loss as measured by OCT can mirror CNS neurodegeneration during MS.

Furthermore, our data suggest an association between retinal vessel loss and brain atrophy in MS that is largely independent of retinal tissue neurodegeneration. In our study, an intra-individual vessel loss of the SVC was associated with both grey and white matter atrophy and a vessel rarefication of the DVC was linked to a decline of grey matter volume, notably independent of alterations of the ganglion cell layer. To the best of our knowledge, our study is the first to analyze connections between the retinal vasculature as measured by OCTA and CNS neurodegeneration as estimated by MRI.

The underlying mechanisms leading to changes of the retinal vasculature and their potential association with CNS neurodegeneration in patients with MS remain unclear. One hypothesis suggests that alterations of the SVC are secondary metabolic effects of retinal ganglion cell loss during MS. The SVC supplies ganglion cells with blood and oxygen. Thus, rarefication of superficial retinal vessels could be a secondary effect of GCIP atrophy due to a lower oxygen demand. In line with this, ganglion cell loss, which is closely related to CNS neurodegeneration, could also result in SVC vessel rarefication. Recently, we could show that superficial but not deep retinal vessel rarefication is evident after acute ON and evolves simultaneously to ON-associated ganglion cell atrophy (10). While this seems to be a possible explanation in eyes with acute ON, it appears less likely to be the underlying mechanism in ON-independent alterations of the retinal vasculature. Firstly, the associations between longitudinal vessel rarefication of the SVC, DVC, and grey matter atrophy remained robust when correcting for ganglion cell loss in our statistical models. Secondly, we detected an association between retinal vessel loss of the DVC and grey matter atrophy. Vessel branches originating from the DVC do not supply retinal ganglion cells and should thus not be affected by ganglion cell atrophy. This suggests that GCIP loss does not entirely explain SVC and DVC thinning and its association with CNS neurodegeneration in patients with RRMS.

On the contrary, ON-independent retinal vessel loss and its association with CNS neurodegeneration could be the consequence of disease-specific alterations of retinal glial cells. Astrocytes, and especially microglia, are thought to be one of the drivers of compartmentalized, chronic, and smoldering inflammation (36). Pathological alterations of astrocytes and microglia are frequently found in brain specimens of patients with MS. They may even occur in CNS areas distant from any inflammatory lesion, for example, in normal-appearing white matter (37, 38). Within the retina, alterations of glial cells are also evident. Histopathological studies in human eyes have revealed a decline of astrocytic Müller cells and an activation of retinal microglia near vessels as a typical finding in patients with MS (11, 39). The blood-retinal-barrier is formed by retinal Müller astrocytes, microglia, pericytes and endothelial cells and modulates the vascular tone and the retinal blood supply (40). Besides MS, retinal vessel loss also occurs during anti-aquaporin 4 antibody positive neuromyelitis optica spectrum disorders. By our group, vessel loss within the SVC has been linked to enhanced serum levels of the glial fibrillary acidic protein (GFAP) suggesting a damage and decline of astrocytes (11, 41). In experimental autoimmune encephalomyelitis, the standard murine model of MS, aquaporin 4 expressing Müller cells are mandatory for the integrity of the blood-retinal-barrier and genetic ablation of aquaporin-4 may result in altered retinal perfusion (42). Based on these findings, we speculate that changes of the retinal vasculature could result from pathological alterations of retinal glial cells. By establishing an association of retinal vessel pathology, an inflammatory immune phenotype (11) and CNS neurodegeneration, our study provides further evidence for this hypothesis.

Our study has different limitations. Firstly, OCTA measurements are device-specific and device-specific effects on the present results and derived conclusions are possible (43). Here, further studies comparing different OCTA devices are needed. Secondly, physiological influences of age and sex on OCTA measures have not been studied in detail yet. It is possible that age affects retinal vessel measures and that the present data might not relevant to younger or older patients. However, we corrected for age, making a purely age-related origin of our findings unlikely. Thirdly, our data and especially the reported longitudinal OCTA measures lack a comparison to healthy subjects. There is very limited data on alterations of the retinal vasculature under healthy conditions and we cannot compare our measures to published findings in the literature. There is, however, substantial literature on longitudinal brain atrophy measures in patients with MS. Our patient cohort revealed a mean cerebral atrophy of 0.4% per year (**Tables 1**, **2**), which is above the rate of healthy cohorts (~0.2%) and in proximity to the threshold which is considered as acceptable under therapeutical conditions (0.4%) (44). This suggests that our patient cohort reveals a rather mild longitudinal cerebral atrophy in the range in published studies. Fourthly, further methodological issues need to be taken into account when interpreting the present findings: It is unclear whether a decline in vessel densities really reflects a true loss of vessel branches or whether it results from hypoperfusion or vasoconstriction. OCTA only provided retinal perfusion patterns but does not report about vessel morphology or integrity. Currently available devices and algorithms do not allow to differentiate whether a reduction in retinal vessel densities reflects vascular constriction, shrinkage of vessels, or true vessel loss. Here, advances in OCTA signal and image processing are needed. Fifthly, we did not screen our patients for the prevalence of arterial hypertension, diabetes or hyperlipidemia in a standardized way since this conditions could affect quantitative analyses of the retinal vasculature (45).

Taken together, our study suggests an association between alterations of the retinal vasculature and CNS neurodegeneration in patients with MS. Our findings provide novel insights into the retinal pathology during MS. If confirmed by other groups, the evaluation of retinal vessels by OCTA could be a novel tool to assess CNS neurodegeneration.

## Data availability statement

The raw data is not accessible to the public due to privacy and ethical considerations. However, upon a reasonable request from a qualified investigator, the data can be shared in an anonymized format.

## Ethics statement

The studies involving humans were approved by ethics committee of the Technical University of Munich, School of Medicine and Health. The studies were conducted in accordance with the local legislation and institutional requirements. The participants provided their written informed consent to participate in this study.

## Author contributions

ER: Data curation, Formal Analysis, Investigation, Methodology, Writing – original draft. TW: Data curation, Writing – review & editing. MB: Data curation, Writing – review & editing. LA: Data curation, Supervision, Writing – review & editing. RW: Data curation, Methodology, Writing – review & editing. CN: Writing – review & editing. AB: Writing – review & editing. VD: Data curation, Writing – review & editing. CM: Methodology, Software, Writing – review & editing. CZ: Methodology, Writing – review & editing. TK: Resources, Writing – review & editing. BH: Conceptualization, Resources, Writing – review & editing. JK: Methodology, Writing – review & editing. MM: Data curation, Formal Analysis, Investigation, Methodology, Resources, Supervision, Writing – review & editing. BK: Conceptualization, Data curation, Formal Analysis, Funding acquisition, Investigation, Methodology, Project administration, Supervision, Writing – original draft.

## References

[1] Petzold A Balcer LJ Calabresi PA Costello F Frohman TC Frohman EM. Retinal layer segmentation in multiple sclerosis: a systematic review and meta-analysis. Lancet Neurol (2017) 16:797–812. doi: 10.1016/S1474-4422(17)30278-8 28920886

[2] Wauschkuhn J Buenrostro GS Aly L Asseyer S Wicklein R Hartberger JM. Retinal ganglion cell loss is associated with future disability worsening in early relapsing remitting multiple sclerosis. Eur J Neurol (2023) 30(4):982–90. doi: 10.1111/ene.15681 36635219

[3] Aly L Havla J Lepennetier G Andlauer TFM Sie C Strauss EM. Inner retinal layer thinning in radiologically isolated syndrome predicts conversion to multiple sclerosis. Eur J Neurol (2020) 27(11):2217–24. doi: 10.1111/ene.14416 32589804

[4] Zimmermann HG Knier B Oberwahrenbrock T Behrens J Pfuhl C Aly L. Association of retinal ganglion cell layer thickness with future disease activity in patients with clinically isolated syndrome. JAMA Neurol (2018) 75(9):1071–9. doi: 10.1001/jamaneurol.2018.1011 PMC6143115 29710121

[5] Saidha S Al-Louzi O Ratchford JN Bhargava P Oh J Newsome SD. Optical coherence tomography reflects brain atrophy in multiple sclerosis: A four-year study. Ann Neurol (2015) 78:801–13. doi: 10.1002/ana.24487 PMC4703093 26190464

[6] Bsteh G Berek K Hegen H Altmann P Wurth S Auer M. Macular ganglion cell-inner plexiform layer thinning as a biomarker of disability progression in relapsing multiple sclerosis. Mult Scler (2021) 27:684–94. doi: 10.1177/1352458520935724 32613912

[7] Mohammadi S Gouravani M Salehi MA Arevalo JF Galetta SL Harandi H. Optical coherence tomography angiography measurements in multiple sclerosis: a systematic review and meta-analysis. J Neuroinflamm (2023) 20:85. doi: 10.1186/s12974-023-02763-4 PMC10041805 36973708

[8] Murphy OC Kwakyi O Iftikhar M Zafar S Lambe J Pellegrini N. Alterations in the retinal vasculature occur in multiple sclerosis and exhibit novel correlations with disability and visual function measures. Mult Scler (2020) 26(7):815–28. doi: 10.1177/1352458519845116 PMC6858526 31094280

[9] Feucht N Maier M Lepennetier G Pettenkofer M Wetzlmair C Daltrozzo T. Optical coherence tomography angiography indicates associations of the retinal vascular network and disease activity in multiple sclerosis. Mult Scler (2019) 25:224–34. doi: 10.1177/1352458517750009 29303033

[10] Aly L Noll C Wicklein R Wolf E Romahn EF Wauschkuhn J. Dynamics of retinal vessel loss after acute optic neuritis in patients with relapsing multiple sclerosis. Neurol Neuroimmunol Neuroinflamm (2022) 9. doi: 10.1212/NXI.0000000000001159 PMC8931743 35301260

[11] Noll C Hiltensperger M Aly L Wicklein R Afzali AM Mardin C. Association of the retinal vasculature, intrathecal immunity, and disability in multiple sclerosis. Front Immunol (2022) 13:997043. doi: 10.3389/fimmu.2022.997043 36439131 PMC9695398

[12] Thompson AJ Banwell BL Barkhof F Caroll WM Coetzee T Comi G. Diagnosis of multiple sclerosis: 2017 revisions of the McDonald criteria. Lancet Neurol (2018) 17:162–73. doi: 10.1016/S1474-4422(17)30470-2 29275977

[13] Colato E Prados F Stutters J Bianchi A Narayanan S Arnold DL. Networks of microstructural damage predict disability in multiple sclerosis. J Neurol Neurosurg Psychiatry (2023) 19:jnnp-2022-330203. doi: 10.1136/jnnp-2022-330203 37468305

[14] Miller DM Thompson NR Cohen JA Fox RJ Hartman J Schwetz K. Factors associated with clinically significant increased walking time in multiple sclerosis: results of a survival analysis of short-term follow-up data from a clinical database. Mult Scler (2015) 21:457–65. doi: 10.1177/1352458514544536 25112816

[15] Knier B Leppenetier G Wetzlmair C Aly L Hoshi MM Pernpeintner V. Association of retinal architecture, intrathecal immunity, and clinical course in multiple sclerosis. JAMA Neurol (2017) 74:847–56. doi: 10.1001/jamaneurol.2017.0377 PMC5822191 28460032

[16] Schippling S Balk L Costello F Albrecht P Balcer L Calabresi P. Quality control for retinal OCT in multiple sclerosis: validation of the OSCAR-IB criteria. Mult Scler (2015) 21(2):163–70. doi: 10.1177/1352458514538110 24948688

[17] Hosari S Hohberger B Theelke L Sari H Lucio M and Mardin CY. OCT angiography: measurement of retinal macular microvasculature with spectralis II OCT angiography - reliability and reproducibility. Ophthalmologica (2020) 243:75–84. doi: 10.1159/000502458 31509842

[18] Wicklein R Yam C Noll C Aly L Banze N Romahn EF. The OSCAR-MP consensus criteria for quality assessment of retinal optical coherence tomography angiography. Neurol Neuroimmunol Neuroinflamm (2023) 10. doi: 10.1212/NXI.0000000000200169 PMC10574825 37813596

[19] Nolan RC Galetta SL Frohman TC Frohman EM Calabresi P Castrillo-Viguera C. Optimal intereye difference thresholds in retinal nerve fiber layer thickness for predicting a unilateral optic nerve lesion in multiple sclerosis. J Neuro-ophthalmol (2018) 38:451–8. doi: 10.1097/WNO.0000000000000629 PMC8845082 29384802

[20] Aytulun A Cruz-Herranz A Aktas O Balcer L Balk L Baroni P. APOSTEL 2.0 recommendations for reporting quantitative optical coherence tomography studies. Neurol (2021) 97:68–79. doi: 10.1212/WNL.0000000000012125 PMC8279566 33910937

[21] Schmidt P Gaser C Arsic M Buck D Forschler A Berthele A. An automated tool for detection of FLAIR-hyperintense white-matter lesions in Multiple Sclerosis. Neuroimage (2012) 59:3774–83. doi: 10.1016/j.neuroimage.2011.11.032 22119648

[22] Dahnke R Yotter RA and Gaser C. Cortical thickness and central surface estimation. Neuroimage (2013) 65:336–48. doi: 10.1016/j.neuroimage.2012.09.050 23041529

[23] Keihaninejad S Heckemann RA Fagiolo G Symms MR Hajnaval JV Hammers A. A robust method to estimate the intracranial volume across MRI field strengths (1.5T and 3T). Neuroimage (2010) 50:1427–37. doi: 10.1016/j.neuroimage.2010.01.064 PMC2883144 20114082

[24] Shattuck DW Mirza M Adisetiyo V Hojatkashani C Salamon G Narr KL. Construction of a 3D probabilistic atlas of human cortical structures. Neuroimage (2008) 39:1064–80. doi: 10.1016/j.neuroimage.2007.09.031 PMC2757616 18037310

[25] Treadway MT Waskom ML Dillon DG Holmes AJ Park MTM Chakravarty MM. Illness progression, recent stress, and morphometry of hippocampal subfields and medial prefrontal cortex in major depression. Biol Psychiatry (2015) 77:285–94. doi: 10.1016/j.biopsych.2014.06.018 PMC4277904 25109665

[26] Wang X Wang X Chou Y Ma J and Zhong Y. Significant retinal microvascular impairments in multiple sclerosis assessed through optical coherence tomography angiography. Mult Scler Relat Disord (2023) 70:104505. doi: 10.1016/j.msard.2023.104505 36621162

[27] Ulusoy MO Horasanli B Isik-Ulusoy S. Optical coherence tomography angiography findings of multiple sclerosis with or without optic neuritis. Neurol Res (2020) 42:319–26. doi: 10.1080/01616412.2020.1726585 32048550

[28] Murphy OC Kalaitzidis G Vasileiou E Filippatou AG Lambe J Ehrhardt H. Optical coherence tomography and optical coherence tomography angiography findings after optic neuritis in multiple sclerosis. Front Neurol (2020) 11:618879. doi: 10.3389/fneur.2020.618879 33384660 PMC7769949

[29] Bostan M Chua J Sim YC Tan B Bujor I Wong D. Microvascular changes in the macular and parafoveal areas of multiple sclerosis patients without optic neuritis. Sci Rep (2022) 12:13366. doi: 10.1038/s41598-022-17344-3 35922463 PMC9349324

[30] Lanzillo R Cennamo G Criscuolo C Carotenuto A Velotti N Sparnelli F. Optical coherence tomography angiography retinal vascular network assessment in multiple sclerosis. Mult Scler (2018) 24:1706–14. doi: 10.1177/1352458517729463 28933233

[31] Lanzillo R Cennamo G Moccia M Criscuolo C Carotenuto A Frattaruolo N. Retinal vascular density in multiple sclerosis: a 1-year follow-up. Eur J Neurol (2019) 26:198–201. doi: 10.1111/ene.13770 30102834

[32] Sinnecker T Oberwahrenbrock T Metz I Zimmermann H Pfueller CF Harms L. Optic radiation damage in multiple sclerosis is associated with visual dysfunction and retinal thinning - an ultrahigh-field MR pilot study. Eur Radiol (2014) 52(1):122–31. doi: 10.1007/s00330-014-3358-8 25129119

[33] Paul F Calabresi PA Barkhof F Green AJ Kardon R Sastre-Garriga J. Optical coherence tomography in multiple sclerosis: A 3-year prospective multicenter study. Ann Clin Transl Neurol (2021) 8:2235–51. doi: 10.1002/acn3.51473 PMC8670323 34792863

[34] Vidal-Jordana A Pareto D Cabello S Alberich M Rio J Tintore M. Optical coherence tomography measures correlate with brain and spinal cord atrophy and multiple sclerosis disease-related disability. Eur J Neurol (2020) 27:2225–32. doi: 10.1111/ene.14421 32602573

[35] Oh J Sotirchos ES Saidha S Whetstone A Chen M Newsome SD. Relationships between quantitative spinal cord MRI and retinal layers in multiple sclerosis. Neurol (2015) 84:720–8. doi: 10.1212/WNL.0000000000001257 PMC4336102 25609766

[36] Hemmer B Kerschensteiner M Korn T. Role of the innate and adaptive immune responses in the course of multiple sclerosis. Lancet Neurol (2015) 14:406–19. doi: 10.1016/S1474-4422(14)70305-9 25792099

[37] Kutzelnigg A Lucchinetti CF Stadelmann C Bruck W Rauschka H Bergmann M. Cortical demyelination and diffuse white matter injury in multiple sclerosis. Brain J Neurol (2005) 128:2705–12. doi: 10.1093/brain/awh641 16230320

[38] Kuhlmann T Ludwin S Prat A Antel J Bruck W and Lassmann H. An updated histological classification system for multiple sclerosis lesions. Acta Neuropathol (2017) 133:13–24. doi: 10.1007/s00401-016-1653-y 27988845

[39] Green AJ McQuaid S Hauser SL Allen R IV Lyness. Ocular pathology in multiple sclerosis: retinal atrophy and inflammation irrespective of disease duration. Brain J Neurol (2010) 133:1591–601. doi: 10.1093/brain/awq080 PMC2877904 20410146

[40] Goldman D. Muller glial cell reprogramming and retina regeneration. Nat Rev Neurosci (2014) 15:431–42. doi: 10.1038/nrn3723 PMC4249724 24894585

[41] Aly L Strauss EM Feucht N Weiss I Berthele A Mitsdoerffer M. Optical coherence tomography angiography indicates subclinical retinal disease in neuromyelitis optica spectrum disorders. Mult Scler (2022) 28(4):522–31. doi: 10.1177/13524585211028831 PMC8961243 34259579

[42] Maisam Afzali A Stuve L Pfaller M Aly L Steiger M Knier B. Aquaporin-4 prevents exaggerated astrocytosis and structural damage in retinal inflammation. J Mol Med (Berl) (2022) 100:933–46. doi: 10.1007/s00109-022-02202-6 PMC9166880 35536323

[43] Trachsler S Baston AE and Menke M. Intra- and interdevice deviation of optical coherence tomography angiography. Klin Monbl Augenheilkd (2019) 236:551–4. doi: 10.1055/a-0747-5333 30919402

[44] Kappos L De Stefano N Freedman MS Cree BA Radue EW Sprenger T. Inclusion of brain volume loss in a revised measure of 'no evidence of disease activity' (NEDA-4) in relapsing-remitting multiple sclerosis. Mult Scler (2016) 22:1297–305. doi: 10.1177/1352458515616701 PMC5015759 26585439

[45] Donati S Maresca AM Cattaneo J Grossi A Mazzola M Caprani SM. Optical coherence tomography angiography and arterial hypertension: A role in identifying subclinical microvascular damage? Eur J Ophthalmol (2021) 31:158–65. doi: 10.1177/1120672119880390 31617414

